# Improvement of the Uniformity and Electrical Properties of Polyaniline Nanocomposite Film by Addition of Auxiliary Gases during Atmospheric Pressure Plasma Polymerization

**DOI:** 10.3390/nano11092315

**Published:** 2021-09-06

**Authors:** Jae-Young Kim, Hyo-Jun Jang, Eunyoung Jung, Gyutae Bae, Soonwon Lee, Choon-Sang Park, Bhumjae Shin, Heung-Sik Tae

**Affiliations:** 1 School of Electronic and Electrical Engineering, College of IT Engineering, Kyungpook National University, Daegu 41566, Korea; jyk@knu.ac.kr (J.-Y.K.); bs00201@knu.ac.kr (H.-J.J.); eyjung@knu.ac.kr (E.J.); doctor047@knu.ac.kr (G.B.); 2 School of Electronics Engineering, College of IT Engineering, Kyungpook National University, Daegu 41566, Korea; impulsive1@naver.com; 3 Department of Electrical and Computer Engineering, College of Engineering, Kansas State University, Manhattan, KS 66506, USA; purplepcs@ksu.edu; 4 Department of Electronics Engineering, Sejong University, Seoul 05006, Korea; hahusbj@sejong.ac.kr

**Keywords:** atmospheric pressure plasma polymerization, auxiliary gas addition, conductive polymer film, conjugated polymer film, plasma polymerization, polyaniline nanocomposites

## Abstract

The morphological and chemical properties of polyaniline (PANI) nanocomposite films after adding small amounts of auxiliary gases such as argon, nitrogen, and oxygen during atmospheric pressure (AP) plasma polymerization are investigated in detail. A separate gas-supply line for applying an auxiliary gas is added to the AP plasma polymerization system to avoid plasma instability due to the addition of auxiliary gas during polymerization. A small amount of neutral gas species in the plasma medium can reduce the reactivity of monomers hyperactivated by high plasma energy and prevent excessive crosslinking, thereby obtaining a uniform and regular PANI nanocomposite film. The addition of small amounts of argon or nitrogen during polymerization significantly improves the uniformity and regularity of PANI nanocomposite films, whereas the addition of oxygen weakens them. In particular, the PANI film synthesized by adding a small amount of nitrogen has the best initial electrical resistance and resistance changing behavior with time after the ex situ iodine (I_2_)-doping process compared with other auxiliary gases. In addition, it is experimentally demonstrated that the electrical conductivity of the ex situ I_2_-doped PANI film can be preserved for a long time by isolating it from the atmosphere.

## 1. Introduction

Plasma polymerization is a process of synthesizing vaporized reactive monomers produced by gaseous plasmas into a polymeric composite [1,2,3,4,5,6,7], and the resulting polymers are generally manufactured on a substrate as a thin film [8,9,10,11,12]. In manufacturing functional polymer films, plasma polymerization methods have several irreplaceable advantages, such as a simple one-step synthesis process, an ecofriendly polymerization process that does not produce chemical waste, a dry process that uses a small amount of monomer, and a room temperature process with low-power consumption [13,14,15,16,17]. Various radicals and reactive species generated through diverse successive interactions with charged particles, vaporized monomers, and neutral gas species remain conserved or react with each other to form crosslinks in atmospheric pressure (AP) plasma polymerization, which uses nonthermal glow discharge at AP. The crosslinking of these reactive monomers grows into polymer nanocomposite groups, which are finally synthesized into a polymer film with a high crosslinking density on the substrate [18,19,20].

Among conductive polymers, polyaniline (PANI) has photo/gas reactivity as well as the good environmental stability and electrical properties [21,22,23,24]. These material properties of polyaniline and the advantages of plasma polymerization are attracting the attention of researchers studying near-future electronic devices such as conductive fabrics, solar devices, displays, and sensors.

Elmas et al. [24] studied the photoresponse behaviors of plasma-deposited PANI films. Various metal ions and an artificial sunlight with energy greater than the band gap of PANI were used for doping of these films. Owing to this, the photoresponse properties of metal- and photo-doped PANI films were dramatically improved.

Liu et al. [25] studied hydrogen chloride (HCl)-doped PANI coated on polyethylene terephthalate (PET) yarn using dielectric barrier discharge under AP. The PANI-coated PET yarn was more conductive than the bare PET yarn. It was also reported that the more PANI was coated on the PET yarn, the better the airbag properties of PET.

Butoi et al. [26] investigated morphological and physicochemical characteristics of PANI film synthesized using a direct current glow plasma. The PANI films were prepared by varying experimental conditions such as power (voltage and current), distance between anode and substrate, substrate inclination angle and aniline monomer temperature.

Pattyn et al. [27] examined the formation of PANI nanoparticles using radio frequency plasma under a low pressure condition. The PANI nanoparticles were characterized by in situ FT-IR and plasma ion mass spectroscopy during polymerization. The generation of partial fragments of the aniline molecule and the interaction of PANI nanoparticles with argon plasma led to increasing the crosslinking of PANI.

Our research group also performed several experimental studies focusing on an efficient AP plasma polymerization process for the formation of PANI nanocomposite [28,29,30]. For the stable generation of glow discharge at AP, a plasma jet device with a dielectric barrier was introduced and three quartz tube jet devices were used as an array to deposit a PANI film on a substrate. Maintaining a stable glow discharge is critical for plasma polymerization because it can supply stable plasma energy to the vaporized monomer molecules. Our research group found that the AP plasma jet (APPJ) array was able to easily maintain stable glow discharge because the discharge current was automatically controlled by the dielectric barrier [28,29,30,31,32]. In addition, the resulting PANI deposited on the substrate was observed in the form of a nanocomposite in which a number of nanofibers and nanoparticles were connected to form a porous network [28,29,30]. However, the only drawback of this apparatus is that a relatively high alternating-current voltage is required to charge and discharge the capacitive structure using the dielectric barrier. The high-voltage driven plasma induces a high-energy state in the plasma medium, resulting in several vaporized monomers becoming hyperactive. They can cause excessive and random crosslinking during polymerization, eventually compromising the uniformity of the polymer film and reducing its quality.

In this study, we propose an additional flow of a small amount of neutral gas toward the substrate as a simple solution to reduce excessive crosslinking by suppressing the reactivity of the aniline monomer species hyperactivated by a high-voltage (HV)-driven plasma. Using the newly developed AP plasma reactor, the hyperactive monomer species compulsively collide with the neutral gas species, many of which lose their energy through inelastic collision with neutral gas particles, resulting in an adequate decrease in their reactivity before reaching the substrate. Preventing excessive and random crosslinking among monomer species induces regular growth of polymer nanocomposites microscopically and improves the uniformity of the polymer film macroscopically.

## 2. Materials and Methods

### 2.1. The Entire AP Plasma Polymerization System

**Figure 1:**
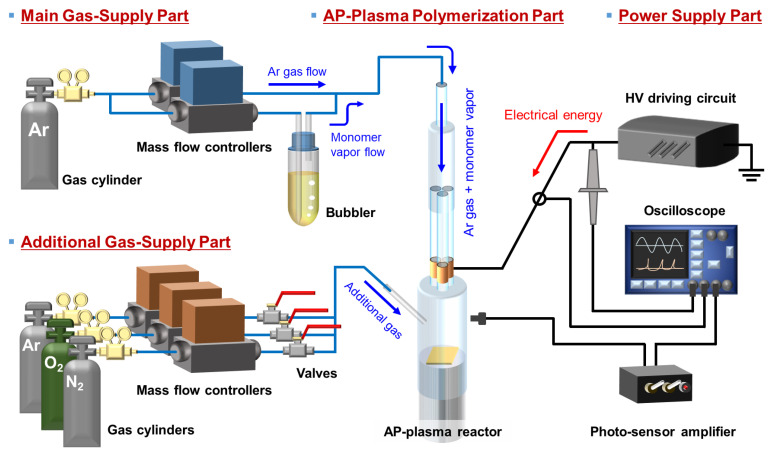
Schematic diagram of the AP plasma polymerization system, equipped with main and additional gas supplies, AP plasma reactor, and HV power supply.

The AP plasma polymerization system used in this study is shown schematically in Figure 1. The basic AP plasma polymerization system, including AP plasma apparatus, monomer vaporizing unit, and gas-supply equipment, has been detailed in [30]. The entire system consists of four major parts: main gas supply, additional gas supply, AP polymerization, and power supply parts. In the main gas-supply part, the gas feed line is divided into two parts for independent control of the gas flows for plasma generation and monomer vaporization. Argon (Ar) gas is used as the discharge gas at a flow rate of 2200 standard cubic centimeters per minute (sccm). A glass bubbler is used to vaporize liquid aniline monomer (MW = 93 g∙mol^−1^, Sigma-Aldrich Co., St. Louis, MO, USA), which is supplied with Ar gas at a flow rate of 500 sccm. The additional gas feed line is split into three to supply different gases of Ar, oxygen (O_2_), and nitrogen (N_2_), and valves are added to prevent the mixing of the different gases. Using this unit, the additional gas is supplied at a flow rate of 50 sccm. All gases used in the study are of high purity (HP) grade with a purity of 99.999%.

Using an inverter-type driving circuit, a sinusoidal voltage with a peak value of 10 kV and a frequency of 27 kHz is applied to the AP plasma reactor. To monitor the electrical characteristics of the generated plasma, the voltage and current waveforms from the powered electrode are monitored using an HV probe (P6015A, Tektronix Inc., Beaverton, OR, USA) and a current transformer (4100, Pearson Electronics Inc., Palo Alto, CA, USA). A fiber optic spectrometer (USB-2000+, Ocean Optics Inc., Dunedin, FL, USA) is used to identify a variety of reactive species generated by the AP plasma polymerization process.

### 2.2. AP Plasma Reactor Capable of Auxiliary Gas Addition

**Figure 2:**
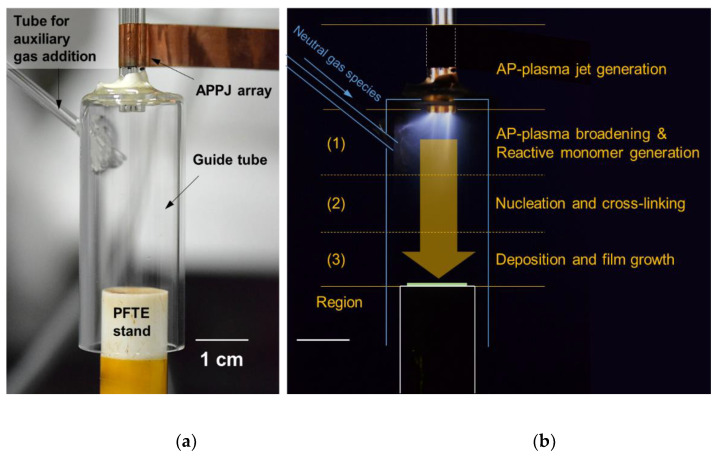
AP plasma reactor: (**a**) labeled photographic image; (**b**) graphical description of the sequence of AP plasma polymerization inside the guide tube.

As shown in detail in Figure 2a, the AP plasma reactor consists of an APPJ array, a guide tube, and a substrate stand. For the APPJ array, three glass tubes arranged in a triangular configuration have copper electrodes to generate plasma jets. All three glass tubes have an outer and inner diameter (ID) of 3 and 1.5 mm, respectively. For the electrode with each glass tube, a copper tape, 10-mm wide, is used and placed 10-mm apart from its end. The space enclosed by the guide tube and the substrate stand acts as a passive chamber for polymerization at AP. The guide tube with a 20-mm ID determines the plasma polymerization area, and the polytetrafluoroethylene substrate stand with a 15-mm diameter is also used as a bluff body, which disrupts the gas flow emerging from the APPJ array, allowing the discharge gas and various reactive products to remain inside the guide tube for a longer time [33,34]. To avoid the adverse effect of plasma generation by adding auxiliary gas, the auxiliary gas is directly added to the middle area of the guide tube through a separate gas line, as depicted in Figure 2a. Using the proposed AP plasma reactor, polyaniline (PANI) nanocomposite films are synthesized by adding small amounts of Ar, O_2_, and N_2_ flows during plasma polymerization.

### 2.3. Ex Situ Iodine Doping Procedure for Electrical Properties of PANI Film

For ex situ iodine (I_2_)doping, PANI film samples deposited on interdigitated electrode (IDE)-patterned silicon (Si) substrates are placed in a container with 2 g of solid I_2_ pellets (Sigma-Aldrich Co., 99.99%) and vacuum sealed using a vacuum sealer packaging machine (VP-5700, Intropack, Pyeongtaek, Korea). After doping for 30 min, the color of the PANI nanocomposite films changed from light beige to dark brown. The width of the IDE substrate is 10.8 µm, and the gap between the adjacent IDEs is 2.54 µm. One IDE substrate consists of 20 pairs of interdigital electrodes, which are made of gold.

### 2.4. Characterization of the Polyaniline Nanocomposite Film

#### 2.4.1. Field Emission-Scanning Electron Microscopy Imaging

The surface morphology, thickness, and vertical alignment of the PANI nanocomposite films on Si substrates were monitored via field emission-scanning electron microscopy (FE-SEM) imaging (SU8220, Hitachi High-Technologies, Tokyo, Japan) with accelerated electrons at a voltage of 3 kV and a current of 10 mA. The samples were coated with conductive platinum to avoid surface charging problems during analysis.

#### 2.4.2. Atomic Force Microscopy Analysis

The surface roughness of the PANI nanocomposite films was investigated using two- and three-dimensional (2D and 3D) topographic images obtained using noncontact mode atomic force microscopy (AFM) (Bruker, NanoWizard II, Ettlingen, Germany) at the Korea Basic Science Institute (KBSI; Busan, Korea). AFM images were generated with 256 × 256 pixels covering an area of 50 × 50 μm, and the scan rate was 1 Hz. All the measurements were obtained under controlled room temperature, and the data were acquired and interpreted with NanoWizard software provided by Bruker.

#### 2.4.3. Attenuated Total Reflection Fourier-Transform Infrared Spectroscopy

The chemical molecular structures of the PANI nanocomposites synthesized under different conditions were examined and compared via FTIR (Vertex 70, Bruker, Ettlingen, Germany) at the KBSI (Daegu, Korea). Attenuated total reflection Fourier-transform infrared (ATR-FTIR) spectra were measured by averaging 128 scans in the range of 650–4000 cm^−1^ at a wavenumber resolution of 0.6 cm^−1^.

## 3. Results and Discussion

### 3.1. Glow Discharge Behaviors during AP Plasma Polymerization

Our research group has previously published details on the formation of polymer nanocomposite films using the AP plasma reactor [28,29,33,34]. The AP plasma polymerization process occurs in the space confined between the guide tube, which is connected to the plasma jet array, and the bluff body, which is the substrate stand. To explain AP plasma polymerization that proceeds sequentially from top to bottom inside the guide tube, the inside of the guide tube can be vertically divided into three, as shown in the regions (1–3) in Figure 2b.

The following processes occur in each region: Region (1): numerous charged particles ejected from the APPJ array into the guide tube expand the plasma region by colliding and exchanging their energy and the vaporized monomer molecules interact with many charged particles and radicals in the plasma medium. Some of them gain energy and are converted into reactive monomer species, which can be observed as a cloudy glow; Region (2): reactive monomer species combine, form nuclei in space, and repeat crosslinking to form small polymer nanocomposites; and Region (3): these small nanocomposites are deposited in the order they reach the substrate to form polymeric nanostructured films. AP plasma polymerization aims to form a uniform polymer nanocomposite film with a constant thickness on a substrate by continuously repeating a series of processes occurring in the regions (1–3).

It is necessary to reduce the reactivity of the monomers hyperactivated by high plasma energy induced by the dielectric barrier structure to improve the uniformity of the polymer film on the substrate by preventing the random growth of polymer nanocomposites. For this purpose, an additional gas line is connected to the guide tube so that neutral gas species are directly supplied to Region (1) of Figure 2b. To reduce the reactivity of hyperactive monomers, Ar, which is the same as the discharge gas, and O_2_ and N_2_, which are relatively safe, even when ionized by plasma, and easy to handle, were selected as auxiliary gases. Therefore, the effects of small amounts of O_2_ and N_2_, which are typical additive gases that are easy to apply, and Ar, a discharge gas, on the formation of PANI nanostructures on the substrate were investigated.

**Figure 3:**
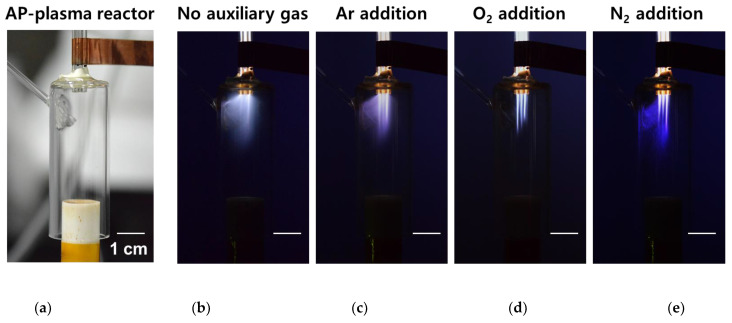
Optical images of glow plasmas during plasma polymerization: (**a**) bright image of the AP plasma reactor and (**b–e**) optical image inside the AP plasma reactor during polymerization; discharge images during polymerization (**b**) without auxiliary gas and (**c**) with the addition of Ar, (**d**) O_2_, and (**e**) N_2_. All auxiliary gases are added at a flow rate of 50 sccm.

Figure 3 shows the plasma behaviors with or without the addition of auxiliary gas through a separate gas line connected to the side of the guide tube. Figure 3a shows a bright image of the AP plasma reactor to verify its configuration and size, and detailed experimental conditions are described in Table 1. Figure 3b shows the discharge behavior during AP plasma polymerization without auxiliary gas. Discharges generated in the APPJ array and plasma plumes were emitted to the guide tube to form a cloudy glow plasma medium. This cloudy glow plasma represents the decomposition of aniline monomer molecules into excited species, fragments, and derivatives by plasma energy, and the cloudy glow emission is mainly caused by the excited aniline species with high reactivity. As the reactive aniline monomer species moved down the guide tube, they were consecutively combined, crosslinked, and nucleated to finally form a PANI nanocomposite film on the substrate.

As shown in Figure 3c–e, the cloudy glow emission was reduced when any of Ar, O_2_, and N_2_ was added during AP polymerization. When the amount of the auxiliary gas was very small, the difference in polymerization characteristics depending on the type of auxiliary gas could not be examined, whereas when the amount of the auxiliary gas was added too considerably, the glow plasma emission became unstable. It was found through repeated experiments that the glow plasma emission and the PANI film properties were rarely changed even if the flow rate of the auxiliary gas of Ar, O_2_, and N_2_ was changed from 30 to 80 sccm. Moreover, in the case of using O_2_ as the auxiliary gas, the glow plasma became unstable when the flow rate of O_2_ was more than 90 sccm. Based on these preliminary works, the flow rate of the auxiliary gas was determined to 50 sccm, similar to the average between 30 and 80 sccm.

When a small amount of neutral gas species was added directly to the region, where reactive monomers were generated (Region (1) in Figure 2b), the reactive monomers lost their energy and became less reactive during collision with neutral gases, resulting in reduced cloudy glow emission. When Ar, the same as the discharge gas, was added, the cloudy glow emission slightly decreased, whereas when O_2_ and N_2_ were added, the cloudy glow was significantly reduced. In particular, when N_2_ was added, a weak blue glow was emitted near the auxiliary gas line, confirming that some neutral N_2_ species participated during the discharge. This phenomenon can also be confirmed by observation of the OES.

**Figure 4:**
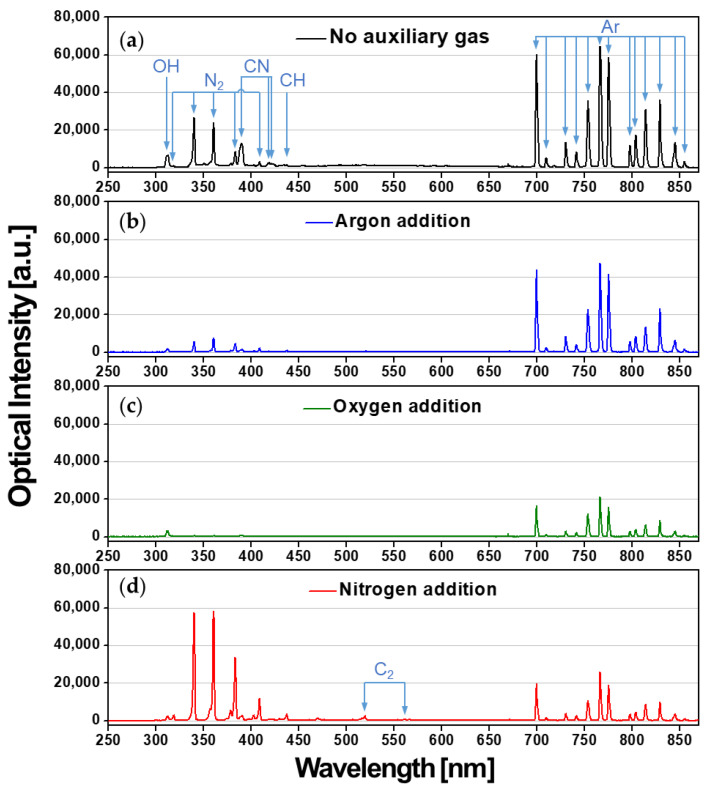
Optical emission spectra measured during AP plasma polymerization (**a**) without auxiliary gas, (**b**) with the addition of Ar, (**c**) O_2_, and (**d**) N_2_.

Figure 4 shows OES peaks from 250 to 870 nm measured in the reactive monomer generation region, indicating that excited N_2_, Ar, and carbonaceous species were present in the plasma medium. As shown in Figure 4a, many of the emission peaks in the ranges of 690–860 nm are for Ar discharge, the peaks at 300–380 nm are for the excited N_2_ species, and there are also several carbonaceous peaks (C–N, C–H, C–C). As shown in Figure 4b–d, the intensity of the Ar peak decreased when any of the gases Ar, O_2_, or N_2_ was added during AP polymerization because additional neutral gas species lowered the intensity of the Ar plasma. In particular, when O_2_, which has better electron affinity than Ar or N_2_, was added, some electrons in the plasma medium were lost because of O_2_ species; thus, Ar discharge was most suppressed (Figure 3d and Figure 4c). As aforementioned, the small addition of N_2_ gas increased the N_2_ peaks, indicating that the neutral N_2_ species participated in the discharge and generated some reactive nitrogen species (RNS). As shown in Figure 4d, the small addition of N_2_ gas also increased the carbonaceous peaks of CH (431.2 nm), and C_2_ (473.5 and 516.3 nm), meaning that more fragments of the aniline monomer existed in the plasma medium.

### 3.2. Changes in Morphological Structure and Chemical Properties of PANI Films

**Figure 5:**
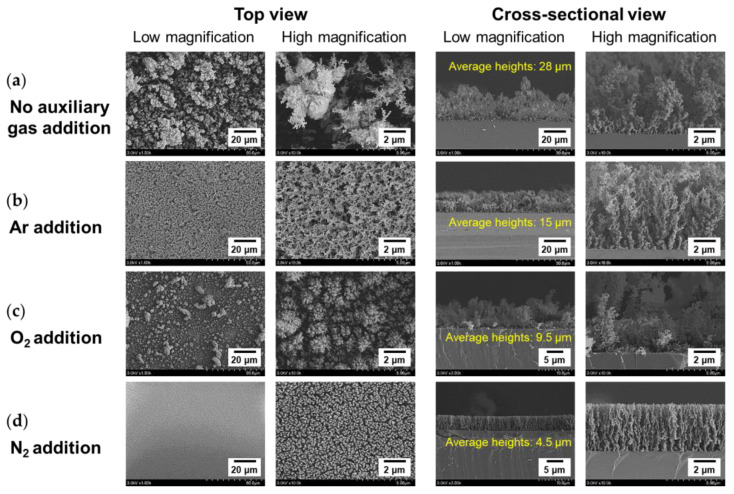
FE-SEM images of PANI films deposited on silicon substrates and polymerized (**a**) without auxiliary gas, (**b**) with the addition of Ar, (**c**) O_2_, and (**d**) N_2_.

The surface and cross-sectional morphologies of PANI films synthesized by adding auxiliary gas during AP plasma polymerization are indicated at different magnifications by the FE-SEM images in Figure 5.

As shown in Figure 5, high magnification FE-SEM images depict that PANI deposited by AP plasma polymerization is a fibrous nanocomposite composed of many nanoparticles and nanofibers, which is in good agreement with our previous studies [28,29]. PANI nanofibers and nanoparticles with a diameter range of 8–20 nm were observed to be linked together to form many irregular cross-linked networks with porosity (Figure 5a), meaning that the nanofibers and nanoparticles were able to be effectively synthesized with the proposed AP plasma reactor. When auxiliary gases (Ar, O_2_, and N_2_) were added during AP plasma polymerization, the heights of the grown PANI films were lower than when no gas was added, indicating that the reactivity of the monomer species or the polymer nanocomposite was effectively reduced by the neutral gas.

When no auxiliary gas was added, PANI nanocomposites grew randomly and rapidly on the substrate due to excessive crosslinking caused by their high reactivity (Figure 5a). As shown in the comparison of Figure 5a,b, when a small amount of inert Ar gas was added, the thickness of the PANI film was reduced to approximately 55%, when compared with that of the PANI film grown in no auxiliary gas addition condition.

Furthermore, when the inert Ar gas was added, the disordered shapes of the PANI nanocomposites were shown to be restored to a regular fibrous morphology, thereby resulting in disappearance of most of the irregular agglomerates. Based on the monitoring of FE-SEM in Figure 5a,b, the addition of inert Ar gas during plasma polymerization can be concluded to induce moderate crosslinking of aniline monomers [35].

Unlike the addition of inert Ar gas, which is chemically stable, the plasmas containing O_2_ and N_2_ usually generate reactive oxygen species (ROS) and RNS, respectively. These generated reactive species caused morphological and chemical differences in the PANI nanocomposites compared to the case where no auxiliary gas was added. When a small amount of O_2_ was added, plasma generation was remarkably suppressed (Figure 4c) and the generated ROS adversely affected not only crosslinking of aniline monomers but also deposition of the PANI film (Figure 5c). In contrast, the addition of N_2_ was more effective in obtaining PANI nanocomposite films with good uniformity and regular microstructure. In this case, the thickness of the resulting PANI film was only 4.5 μm, but the vertical alignment of nanocomposites and the entire film uniformity were improved considerably (Figure 5d). The auxiliary addition of N_2_ gas contributed to interfering with excessive crosslinking and rapid growth of PANI nanocomposites, so that they were well-aligned vertically on the substrate and exhibited dense surface morphology. As a result of the observation of the different fibrous microstructures of the PANI films by FE-SEM, it is confirmed that the addition of auxiliary gases can properly control the degree of crosslinking of PANI nanocomposites [35].

**Figure 6:**
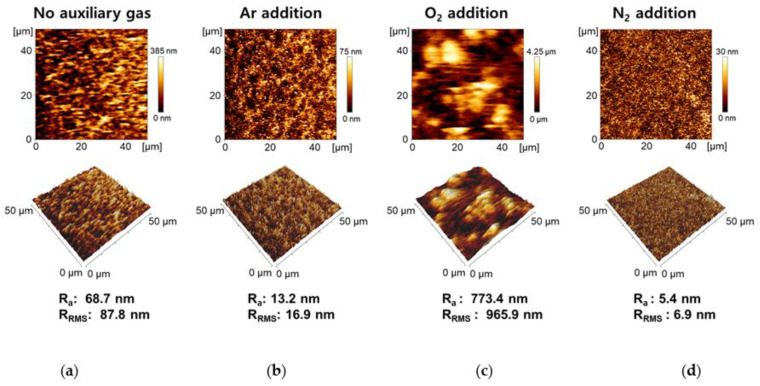
2D and 3D AFM images with the roughness of PANI films prepared (**a**) without auxiliary gas, (**b**) with the addition of Ar, (**c**) O_2_, and (**d**) N_2_.

Figure 6 shows the changes in the 2D and 3D AFM images based on the surface roughness of PANI nanocomposite films on a silicon substrate when various auxiliary gases were added during AP plasma polymerization. These 2D and 3D AFM images provide information about the average surface roughness (R_a_) and root mean square roughness (R_RMS_) in the 50 × 50-µm region of the PANI films. Compared to PANI films synthesized without the addition of auxiliary gas during polymerization, when Ar and N_2_ are additionally used, the surface roughness of the films was reduced to approximately 1/5 and 1/12, respectively, resulting in smooth and uniform PANI nanocomposite films. Conversely, the addition of a small amount of O_2_ increased the surface roughness by 11 times, resulting in an irregular and disordered PANI film surface. When 50 sccm of O_2_ and N_2_ flows were added during AP plasma polymerization, respectively, the surface roughness of the resulting PANI films differs by 140 times.

**Figure 7:**
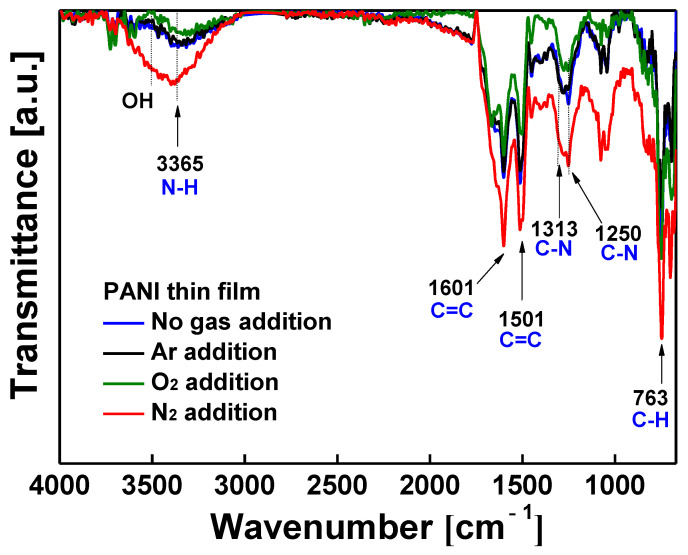
Comparison of ATR-FTIR spectra of PANI films synthesized with and without auxiliary gas addition (Ar, O_2_, and N_2_).

Figure 7 depicts the ATR-FTIR spectra of the PANI films, which show the characteristic peaks of PANI polymeric nanocomposites as follows: benzenoid stretching vibration (1501 cm^−1^), quinoid ring stretching vibration (1601 cm^−1^), C–H out-of-plane deformation from the aromatic ring (763 cm^−1^), C–N stretching vibrations (1250 and 1313 cm^−1^), and N–H stretching vibration (3365 cm^−1^). The ATR-FTIR peaks of the PANI film when a small amount of Ar was added or when Ar was not added during AP plasma polymerization were almost identical. This result indicates that the morphology of the PANI film changed, but its chemical composition did not change when neutral Ar gas was added. The addition of the same gas species as the discharge gas to lower monomer reactivity can be a simple solution when it is necessary to improve the film uniformity while maintaining the chemical properties of the polymer.

On the other hand, the intensity of ATR-FTIR peaks changed when small amounts of O_2_ and N_2_ were used as auxiliary gases, meaning that the generated ROS and RNS caused not only morphological changes but also chemical differences in the PANI nanocomposite. When N_2_ was added during AP plasma polymerization, most of the main peaks on ATR-FTIR were increased, whereas when O_2_ was added, they were decreased. In particular, the intensity of the C−N bond absorption peaks (1250 and 1313 cm^−1^) significantly increased in the PANI nanocomposite film with the addition of a small amount of N_2_, reflecting the relationship between C−N bonds and electrical properties of the PANI nanocomposite film. The C−N bond is closely connected to the electrical conductivity for the proton acid, which is preferred to the N of the quinone ring [36,37]. The increase in conjugated bonds caused by adding N_2_ is expected to improve the π–π stacking of intermolecular polymer chains, thereby enhancing carrier mobility and good electrical conductivity.

### 3.3. Electrical Properties of the Ex Situ Iodine Doped Polyaniline Film

In order to utilize the conjugated polymer film as an electrical material such as an electrode or a transducer, it is important not only to synthesize the conjugated polymer film but also to impart electrical properties to the film. A common method to make a conjugated polymer film conductive is to dope a halogen element such as HCl and I_2_ as a proton donor (electron acceptor) [24,38,39,40]. In particular, the ex situ I_2_ doping process, in which a doping step is performed after synthesizing a polymer film, is widely used to test the conductivity of polymer films due to its low cost and simplicity of the process [41,42,43,44]. When I_2_ is used as a dopant, the electrons in the double bonds of the PANI backbone are transferred to the iodine, leaving the units of the polymer chain positively charged, thus resulting in an imbalanced electron arrangement that makes the PANI film conductive [45]. However, the conductivity of the ex situ I_2_ doped polymer film is generally strongly affected by the ambient humidity. Therefore, it is noted that this doping method has the drawback of gradually increasing electrical resistance with time [46].

The chemical and material properties of ex situ I_2_-doped PANI films have already been studied in detail using elemental analysis instrument (GPC, XRD, XPS, and ToF-SIMS) [47]. As a result of elemental analysis, the content of oxygen-containing functional groups and C=C double bonds in the PANI nanocomposite slightly increased, and the CN and CH bonds slightly decreased with increasing doping time. However, no significant changes were found in the chemical bonding and composition of the PANI film before and after ex situ I_2_ doping.

**Figure 8:**
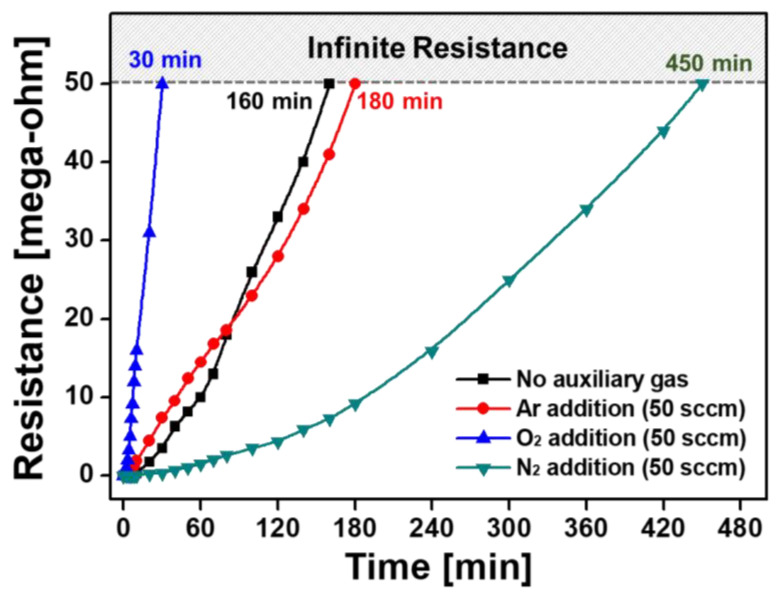
Changes in the electrical resistance of ex situ I_2_-doped PANI films on IDE substrates synthesized with and without auxiliary gases (Ar, O_2_, and N_2_).

Figure 8 shows the changes in the resistance of ex situ I_2_-doped PANI films on IDE substrates relative to exposure time under ambient air. The measurement limit of the resistance of I_2_-doped PANI film is 50 MΩ, and if it is exceeded, it is termed infinite resistance. When no auxiliary gas was added, the initial resistance of the ex situ I_2_-doped PANI film was measured to be 30 kΩ, and the resistance gradually increased in the atmosphere and reached the measurement limit, 50 MΩ, after 160 min. When Ar was added as the auxiliary gas, the initial resistance was good at 8 kΩ, but the resistance change was similar to that when no auxiliary gas was used, and the measurement limit was reached in 180 min.

When O_2_ was used as the auxiliary gas, the initial resistance was measured to be the highest—33 kΩ—and the resistance increased rapidly in the atmosphere and reached the measurement limit in only 30 min. In contrast, when N_2_ was auxiliary added, the initial resistance was excellent at 7 kΩ and the resistance also increased gradually in the atmosphere and had electrical properties for 450 min until it reached the measurement limit. This significant difference in resistance stability is related to the density and regularity of PANI nanocomposites, as shown in the FE-SEM images (Figure 5). The PANI nanocomposite film synthesized with the addition of N_2_ was changed to be very dense and uniform with good vertical alignment, so it was less affected by hydration in the ambient air, improving the resistance stability. Conversely, in the PANI film synthesized by adding O_2_, the PANI nanocomposite was not vertically aligned at all and formed irregular and sparse polymeric networks of nanoparticles and nanofibers. It was confirmed that the characteristics of resistance change according to the vertical growth pattern of these nanocomposites were in good agreement with our previously reported results [46]. The resistance of the PANI film polymerized with N_2_ addition could be measured for another 5 h after the resistance of the PANI film without auxiliary gas reached the measurement limit. Note that the period during which the PANI film exhibited electrical properties was measured 2.8 times longer. Electrical conductivity was improved because the C–N bonds, which are related to the conductivity of the conjugated polymer, were relatively increased by RNS generated in the plasma medium due to N_2_ addition (Figure 7).

**Figure 9:**
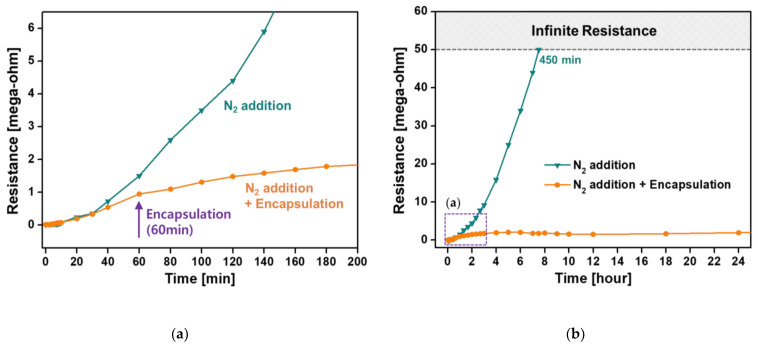
Comparison of the electrical resistance of ex situ I_2_-doped PANI films with and without encapsulation: (**a**) resistance changes in initial 3 h and (**b**) resistance changes in 24 h. Encapsulation test proceeds with PANI films polymerized with N_2_ gas addition.

As aforementioned, many conductive polymer materials change their electrical properties due to water inside or on the surface of their nanocomposite [47,48,49]. Therefore, the conductive polymer layer should be encapsulated with a sealing material to prevent the access of moisture and oxygen to ensure electrical conductivity [50,51,52]. The encapsulation test was conducted with the PANI film synthesized by adding a small amount of N_2_ during AP plasma polymerization, which has the best electrical conductivity. When the electrical resistance reached 1 MΩ at 60 min, the PANI film area of the IDE substrate was isolated from the external atmosphere with polyimide tape (Kapton^®^ tape, DuPont, Wilmington, DE, USA) and high-elasticity sealing film (PARAFILM^®^ M, Bemis Company, Neenah, WI, USA). As a result, the resistance value of the PANI film increased very slowly and stabilized to 1.2–1.6 MΩ by 24 h (Figure 9a,b).

The resistance value of the encapsulated PANI film required 3 days to reach 2 MΩ from 1 MΩ and a total of 10 days to reach the measurement limit of 50 MΩ. We attribute this long-term resistance change to the incomplete encapsulation method used in this study, which used a polyimide tape and elastic sealing film to block the outside atmosphere. If more advanced and accurate encapsulation techniques can be used, the resistance value can remain unchanged. For polymer display applications, a future detailed parametric study will be conducted to measure the electrical properties of plasma polymers created using the proposed AP plasma polymerization.

## 4. Conclusions

In summary, the effect of the addition of auxiliary gas during AP plasma polymerization on the changes in the morphological and electrical properties of PANI films was investigated. The purpose of adding small amounts of gas is to prevent excessive crosslinking by reducing the reactivity of aniline monomers overactivated by high plasma energy, thereby securing the uniformity and regularity of the PANI nanocomposite films. For this reason, the auxiliary gas was directly added to the middle area of the guide tube through a separate gas line. When N_2_ was added as the auxiliary gas, the surface roughness of the PANI nanocomposite film was reduced by more than 10 times compared with that of the conventional one with no gas addition; thus, the film’s uniformity was significantly improved. In addition, the electrical conductivity of the ex situ I_2_-doped PANI film was significantly improved when N_2_ was added. Therefore, adding a small amount of auxiliary gas is a simple method for changing the properties of conjugated polymers without affecting delicate AP plasma polymerization conditions. To use the conjugated polymer nanocomposite film as a transducer (i.e., detecting layer) of a gas sensor, it may be necessary to control the surface roughness to further facilitate the entrapment of gas molecules. For this purpose, the proposed AP plasma polymerization method that appropriately adds an auxiliary gas to the plasma medium can be a simple and effective solution.

## Figures and Tables

**Table 1:** Summarized experimental conditions for AP plasma polymerization with the addition of auxiliary gas.

Experimental Conditions		AP Plasma Reactor
Device Configuration	Discharge type	Glass tube array
	Electrode type	Single electrode
	Electrode material	Copper tape
	Inner diameter of guide tube	20 mm
	Diameter of substrate stand	15 mm
Driving Conditions	Voltage waveform	Sinusoidal
	Plasma initiation voltage (V_p_)	12 kV
	Plasma driving voltage (V_p_)	10 kV
	Driving frequency	27 kHz
	Averaged power dissipated (P_RMS_) ^1^	70 W
Gas Conditions	Discharge and monomer carrier gas	Ar (HP grade: 99.999%)
	Flow rate for AP plasma discharge	1500 sccm
	Flow rate for aniline monomer vapor	500 sccm
	Auxiliary gas species	Ar, O_2_, N_2_ (HP grade)
	Flow rate for auxiliary gas	50 sccm
	Polymerization process time	30 min

^1^ Average power of the AP plasma reactor is calculated as P_RMS_ = V_RMS_ × I_RMS_.

## Data Availability

Not applicable.
